# Auscultation in Pulmonary Tuberculosis

**Published:** 1915-07

**Authors:** Arch Elkin

**Affiliations:** Grand Opera House, Atlanta, Ga.


### AUSCULTATION IN PULMONARY TUBERCULOSIS.

By Arch Elkin, M. D.

It would be quite hackneyed to refer to the importance of auscultation in the field of Physical Diagnosis, particularly when dealing with the subject of this meeting.

We are all aware that the finer points of diagnosis in the early cases of pulmonary tuberculosis depend almost entirely upon what we hear and the correct interpretation of the sounds produced, so that in doubtful cases the last court of appeals, so to speak, is the stethoscope in the hands of one skilled in its use, and one willing to make repeated and thorough examinations. Only under these conditions should auscultation be considered important. Certain it is that its importance is only augmented to a degree of real worth when properly and persistently used. This is true to the extent that only by this means are we able to make a diagnosis of early tuberculosis in the absence of laboratory and other clinical evidence. The majority of early cases give no definite information on inspection, palpation and percussion and in many of them the clinical manifestations and history present an obscure picture. Indeed, the auscultatory signs are often vague.

This type of case requires diligence and patience on the part of the examiner to arrive at any definite conclusion, and I believe the neglect of an early auscultatory sign, regardless of how seemingly unimportant, many times results months later in the perfectly apparent case. The fault is not with auscultation but due to the lack of attention paid obscure abnormal signs.

It is probably true that every case presenting obscure signs which persist for a definite period and which cannot be accounted for in any other way, means pulmonary tuberculosis. The interpretation, then, of sounds, early obscure sounds, is the paramount phase of auscultation and only through the correctness of this interpretation is auscultation made important.

What then are the signs we should consider and how are we to differentiate them? A clear knowledge of the different types of breathing is essential since we must recognize variation in the expiratory note, a sign always mentioned but seldom regarded when occurring alone. We know that in vesicular breathing there is a great similarity of tone in the inspiratory and expiratory murmur and that these murmers so merge that scarcely any variation is noted, yet with bronchial breathing the expiration becomes harsh, loud, higher in pitch and considerably lengthened. The simple differentiation then of vesicular breathing and bronchcial breathing enables us to recognize the prolonged expiratory note occurring over an area removed from the larynx, trachea, etc. In tuberculosis we know this most often occurs in the apices, so that careful study of respiration will often reveal this abnormality—obscure yet important. The fact that so many adult lungs appear to be abnormal either from X-ray examination or from post-mortem does not alter this fact, since this physical appearance is from an entirely different source—the infiltration of fibrous tissue. I mention this because many lungs may have this appearance resulting from a previous inflammatory process and the breath sounds be practically normal.

The so-called harsh breathing occurring over vesicular structure is practically always attended by a prolonged expiration, and this sign persisting in a given area over an extended time has a real signficance. This has been referred to as the first physical sign, but its importance is often unappreciated.

Carefully studied whispered fremitus gives us the best idea as to the extent of the infiltration. When percussion has failed to show an altered note; when palpation fails to produce significant vibrations, the delicacy of the whispered voice will often trace the lesion to its border if we persistently use it and become accustomed to its value.

The border-line cases present the opportunity to use auscultation to its best advantage and if we search repeatedly for these variations our diagnostic acumen will be quickened to a high degree. Constant comparison, however, of the different breath sounds is the only means by which we can recognize these small variations, and no matter how many chests one may examine, unless he with each examination compares what seems to be abnormal with what he knows to be normal, he soon fails to recognize either.

I have purposely avoided mentioning the signs produced in an obvious case since auscultation in these cases serves as does inspection, palpation and percussion, one to corroborate the other. I believe the real value of this procedure is in the vague signs mentioned above.

Grand Opera House, Atlanta, Ga.

---

